# Congenital cystic eye associated with a low-grade cerebellar lesion that spontaneously regressed

**DOI:** 10.1186/1471-2415-14-80

**Published:** 2014-06-17

**Authors:** Maria Giuseppina Cefalo, Giovanna Stefania Colafati, Antonino Romanzo, Alessandra Modugno, Rita De Vito, Angela Mastronuzzi

**Affiliations:** 1 Haematology-Oncology Department, Bambino Gesù Children’s Hospital, IRCCS, Rome, Italy; 2 Neuroradiology Department, Bambino Gesù Children's Hospital, IRCCS, Rome, Italy; 3 Pediatric Ophthalmology Division, Bambino Gesù Children's Hospital, IRCCS, Rome, Italy; 4 Ocularistica Italiana of Rome, Rome, Italy; 5 Unit of Pathology, Bambino Gesù Children's Hospital, IRCCS, Rome, Italy

**Keywords:** Congenital cystic eye, Anophthalmia, Brain lesion, Spontaneous regression, Magnetic resonance imaging

## Abstract

**Background:**

Congenital cystic eye is an exceedingly rare ocular malformative disease, originated from the failure in the invagination of the optic vesicle during the fetal period and it can be associated with other ocular and non-ocular abnormalities. Diagnosis is based on clinical, radiological and histological features.

**Case presentation:**

We report a case of a congenital cystic eye associated with a cerebellar lesion accidentally detected at magnetic resonance imaging. Biopsy of the mass has not been performed due to parental rejection. Based on radiologic features and absence of clinical signs, a low-grade glioma diagnosis was hypothesized, but histological characterization was not obtained. Follow-up neuro-imaging 6 months after diagnosis showed that intracranial lesion spontaneously regressed without any treatment.

**Conclusion:**

Our report stresses the importance of early MRI in children with ocular malformations, in order to detect associated intracranial defects, also of non-malformative origin. Additionally, we debate the clinic-radiological features of the intracranial lesions that could allow a wait-and-see policy. We also recommend a strict clinical and neuro-imaging follow-up for these lesions. Finally, biological mechanisms at the base of spontaneous regression of the brain lesions are discussed.

## Background

Congenital cystic eye [1] is a very rarely recognized ocular malformation with fewer than 40 cases reported. The condition derived from the partial or complete arrest in the invagination of the primary optic vesicle during the fourth week of gestation [2]. At the best of our knowledge, congenital cystic eye can be defined as a non-hereditary disorder of unknown origin. The disorder is most commonly unilateral, but bilateral congenital cystic eyeball has been recognized by Sacks et al. [3]. They investigated the central visual pathways in bilateral congenital cystic eye and evidenced that the intracranial portion of one optic nerve represented a remnant of the optic stalk and no chiasm was found. The malformation is usually present at birth or may become apparent later in childhood. The congenital cystic eye may be cystic or solid and the cyst may vary in size in relation to the patency of the stalk, and may be single or multiple. Connective tissue lined by neuroglial material composes the wall of the congenital cystic eye. The ocular structures derived from surface ectoderm, as lens or cornea, is lacking and the extra-ocular muscle surrounding the malformation may be normal or defective. Congenital cystic eye may be isolated or associated with intra or extra-ocular malformations [4]. The most common intraocular malformation described in association with congenital cystic eye is microphthalmia with cyst [5], as a rare entity cataloged on the spectrum of colobomatous eye disorders. Persistent hyperplastic primary vitreous in the fellow eye [6], dermal appendages [7], eyelid coloboma have been also recognized in association with congenital cystic eye. Systemic associations [8] include saddle nose, facial clefting, nostril malformation, choanal atresia, malformation of the sphenoid bone and other neurological abnormalities. Brain magnetic resonance imaging (MRI) [9] is not only useful in the diagnosis and management of this rare entity, but it can also be helpful in diagnosing associated brain abnormalities. Differential diagnosis between this malformation and different cystic malformations and masses of the orbital cavity and eyeball is often very difficult also for expert clinicians. Surgical exploration and histopathology are also crucial for diagnosis.

We here report a rare case of a congenital cystic eye associated with a glial cerebellar lesion accidentally detected at magnetic resonance, that spontaneously regressed during follow-up. Finally, we also debated the biological mechanisms at the base of spontaneous regression of the brain lesions.

## Case presentation

**Figure 1:**
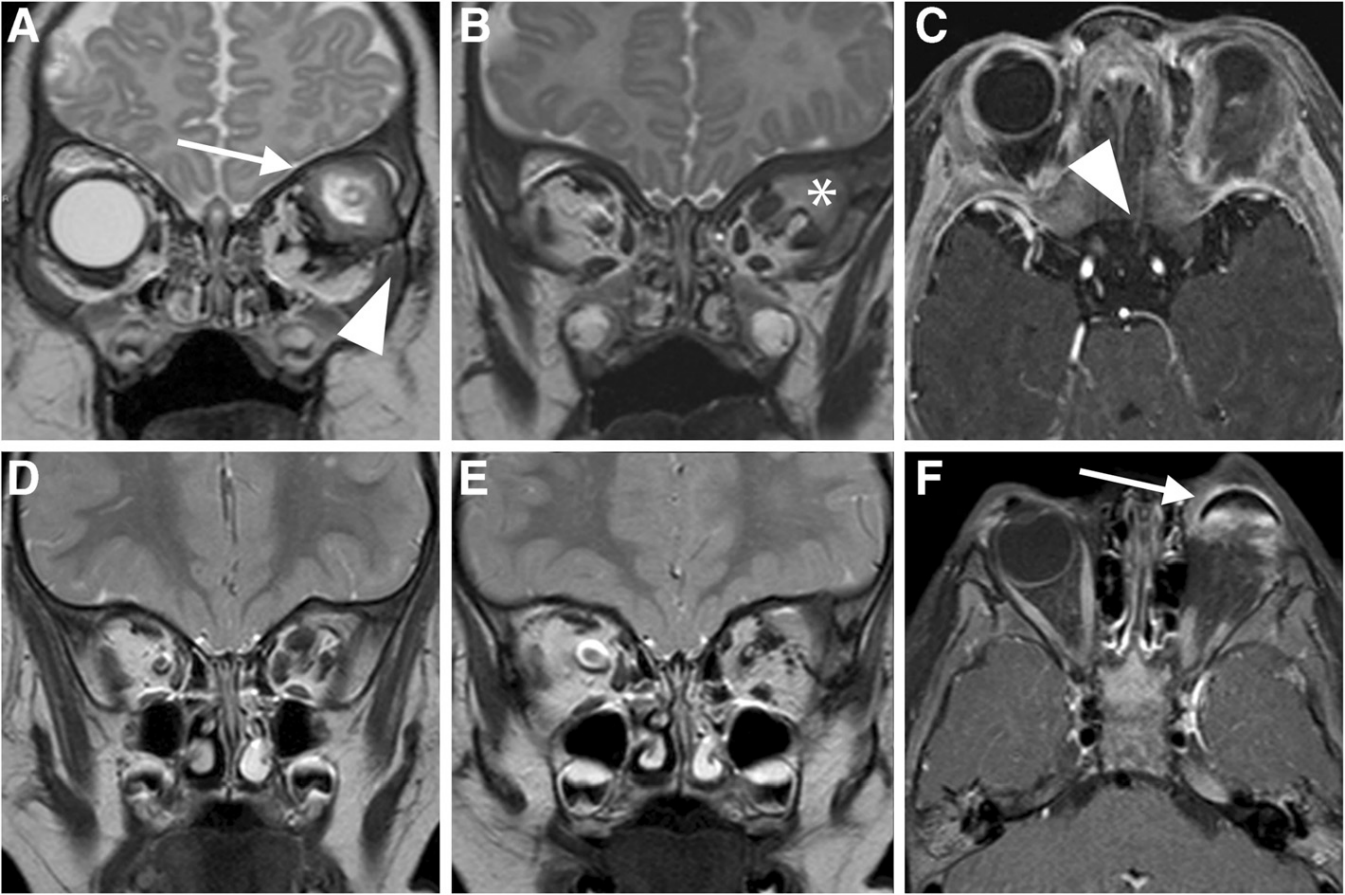
**Pre-operative (A-C) and post-operative (D-F )orbit MRI study.** Coronal T2w images **(A-B)** show a complex mass in the upper-outer and anterior portions of the left orbit with a predominantly extraconal engagement. The mass consists of a well-defined anterior cystic component (white arrow) with signal intensity similar to vitreous/cerebrospinal fluid (CSF) and a posterior component more hyperintense than the muscle signal. No normal orbital contents such as extra-ocular muscles or globe can be identified in the anterior portions of the orbit. Axial post-gadolinium fat saturated T1w image **(C)** shows heterogeneous enhancement of the posterior soft tissue mass. No normal orbital contents such as extraocular muscles or globe can be identified in the anterior portions of the orbit. Notice the bone defect of the lateral wall of the orbit (**A**, arrowhead) and the left optic nerve atrophy (**C**, arrowhead). Post-operative Coronal T2w MRI images **(D-E)** disclose a partial reduction of the complex mass in the left orbit; following gadolinium administration, the residual lesion shows a dyshomogeneous enhancement (axial post-gadolinium fat saturated T1w image, **F**); notice the presence of a left ocular prosthesis (**F**, white arrow).

**Figure 2:**
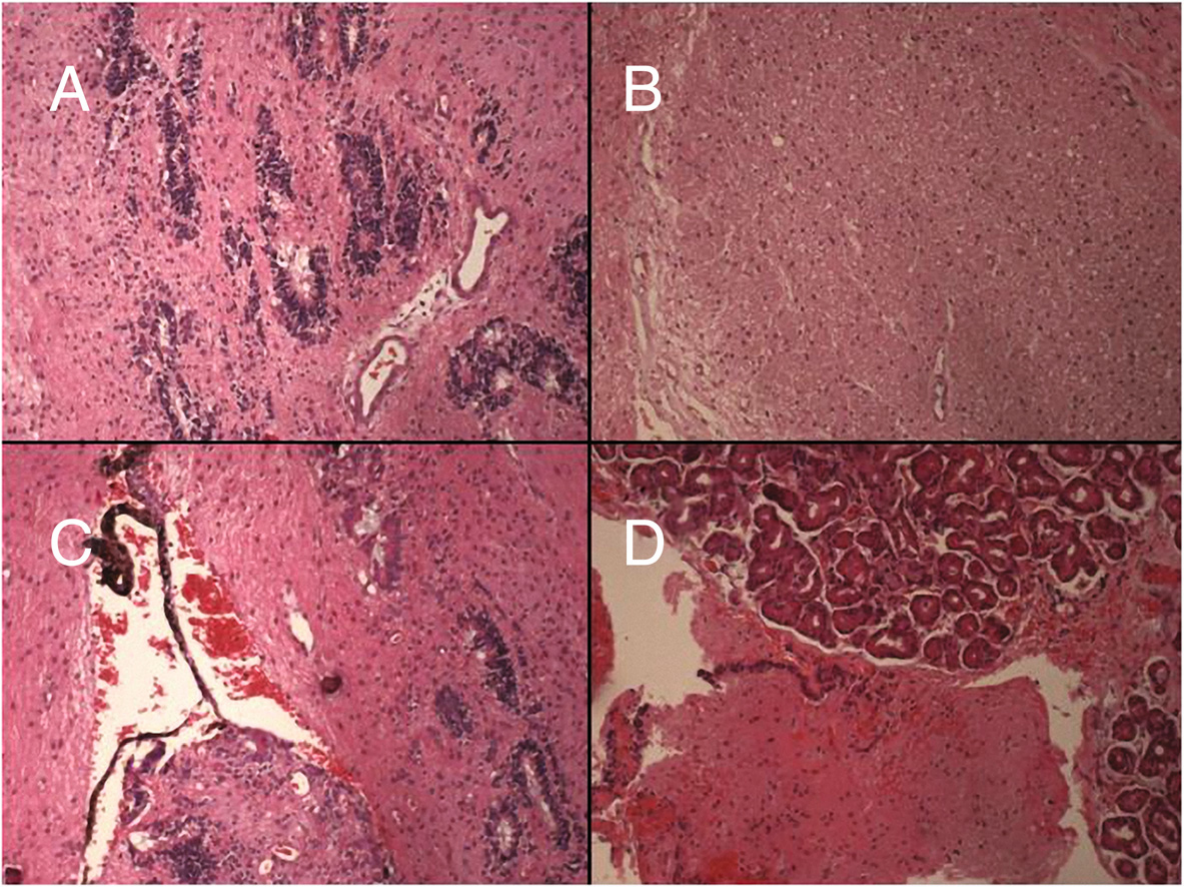
**Histopathological characterization. A-D**. Hematoxylin-Eosin-Stained Histopathological Sections (original magnification x10): the cystic wall was formed by fragments of a fibrous tissue containing strands of pigmented retinal epithelium. Some islands of glial tissue composed by glial fibers intermixed with stellate astrocytes with moderate eosinophilic cytoplasm were also present. No cellular atypia, mitotic activity or necrosis was evident. No tissue from other germ cell layer was seen.

A 6-month-old child was referred to our Ophthalmology Department for a congenital unilateral cystic orbital mass, noticed at birth. Ophthalmologic examination revealed a left empty socket and a cystic lesion protruding from the left orbit, stretching upper eyelids. No ocular structures could be identified. The child didn’t present cafè-au-lait spots, specific cutaneous lesions or Lisch nodules in the iris. Patient was tested by array comparative genomic hybridization, without evidence of genomic abnormalities: in particular, SOX2/PAX6 mutations were excluded and karyotype was normal. On the ultrasonography, no globe was disclosed in the left cavity. Brain and orbit MRI suggested a non-infiltrative extraconal superior lesion (Figure 1) that variably contained solid and fluid mixed components. No other intracranial alterations were evident. The high risk of rupture of the cyst, caused by the thin wall and the adherence to connective tissue of the peri-orbita, was prevented by aspiration of the fluid, but a radical dissection from the surrounding structures was not easy, so the mass was partially excised. Histopathological examination of the cyst disclosed a complex structure primarily composed of neuroglial tissue, as described in Figure 2. Immunohistochemical staining revealed positivity for neuron specific enolase (NSE) and synaptophysin. These findings were coherent with diagnosis of congenital cystic eye. Follow-up brain MRI, performed one year after surgery (Figure 3A-C), disclosed a focal altered signal consistent with a superficial, well-described solid lesion in the right cerebellar cortex, 8 mm in maximum diameter, hypointense on T1-weighed and hyperintense on T2-weighed images, with mild and inhomogeneous enhancement after gadolinium administration. In the lesion site, no signal alterations using DWI sequences could be detected. Based on the absence of clinical signs and considering the radiologic features in pediatric setting, an etero-formative lesion of glial origin was suspected. Biopsy was recommended, but parental consent was not obtained. Therefore, a wait-and-see policy was forcedly applied. The parents also refused a brain MRI after 1 month. Surprisingly, brain MRI performed 6 months after the diagnosis of the intracranial lesion demonstrated a complete regression without any treatment (Figure 3). Subsequent neuro-imaging follow-up confirmed this spontaneous involution and the child currently remains disease-free 24 months after diagnosis.

**s:**
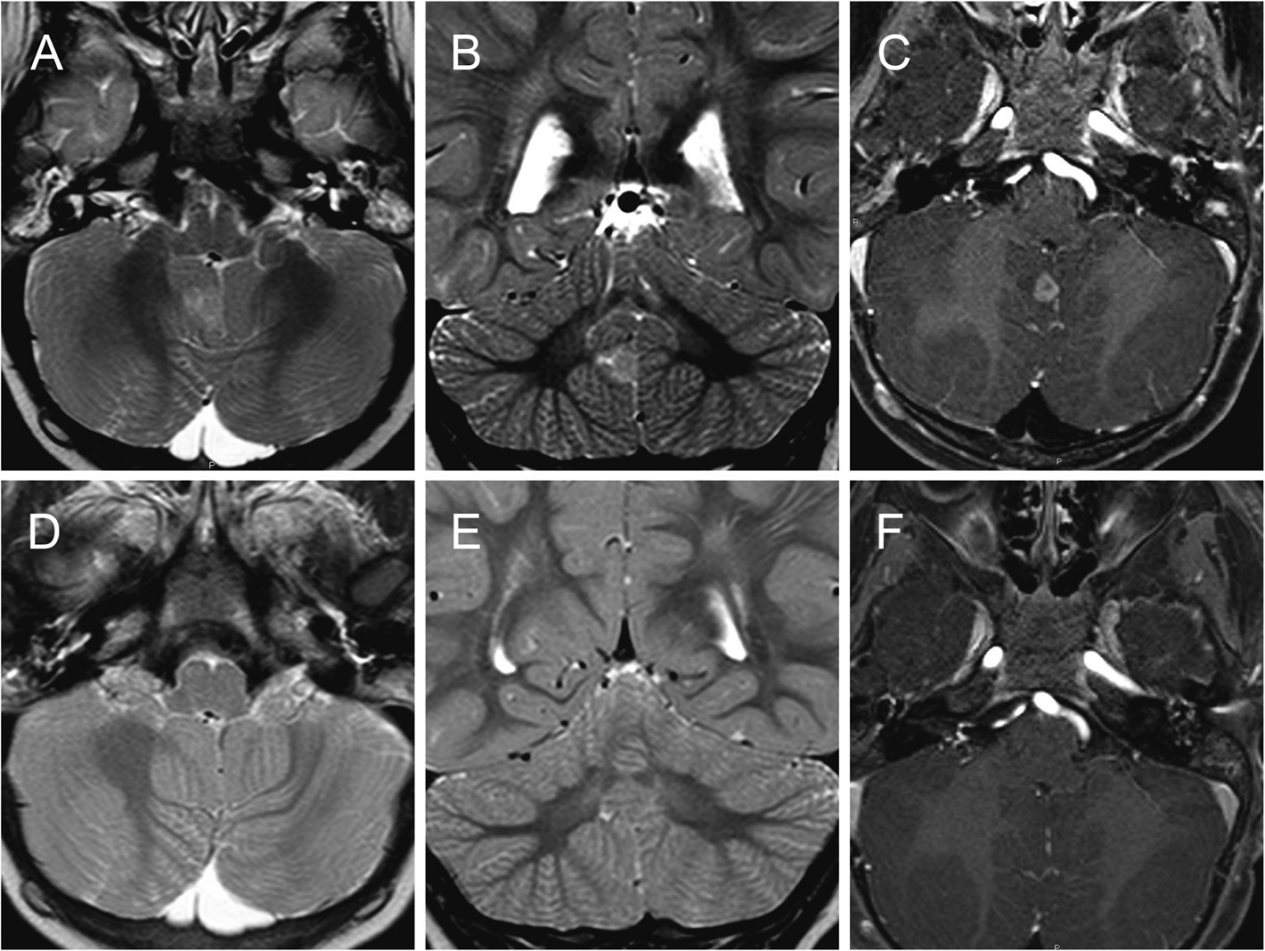
**Evolution of the cerebellar lesion on brain MRI study.** Axial **(A,D)** and coronal **(B,E)** T2 weighted MRI;. Axial **(C,F)** post-contrast T1-weighted MRI: small pseudonodular lesion on the mesial side of the right cerebellar hemisphere. The lesion was hyperintense on T2-dependent sequences **(A,B)** and with mild enhancement after gadolinium administration **(C)**. Follow-up MRI performed after 6-months revealed complete disappearance of the cerebellar lesion **(D-E-F)**.

## Discussion

To our knowledge, less than 40 cases of congenital cystic eye, also termed “anophthalmos with cysts”, have been published to date. Duke-Elder [10] reviewed the ophthalmic literature from 1880 to 1960 and only 16 cases have been recognized. Since then, less than 20 other cases have been reported in literature, as recognized by Guthoff et al. [11]. Also two single cases described by Dash et al. [12] and Shukla et al. [13] were not evaluated histopathologically.

This rare disembriogenetic disorder results from a defect in the invagination of the primitive optic nerve vesicle, causing the incomplete evolution of the ectodermal elements into the mature eye tissues between the 2-mm and 7-mm stages of fetal development. The etiology of the cystic eye remains unclear; genetic investigations performed in few cases have not yielded any peculiar defects [14,15], as in our case. Congenital cystic eye has been rarely recognized in association with other intracranial non-ocular alterations [16], as agenesis of corpus callosum, midbrain deformity, basal encephalocele and microcephaly. The definition of “cranial ectodermopathy” [17] has been coined in order to unify this constellation of abnormalities, predominantly of malformative origin. To our knowledge, the association between congenital cystic eye and etero-formative lesions of the central nervous system has been exceptionally described: Mehta et al. [18] reported the case of a 13-year-old girl affected by a congenital cystic eye in association with ectopic glial tissue in the subependymal region of the left ventricle. No glial lesions in the cerebellum associated with congenital cystic eye have been previously recognized.

Diagnosis is based on clinical, imaging and histopathological features. Newer imaging modalities, as prenatal magnetic resonance imaging, could reveal characteristic findings of this condition at or even prior to birth, as suggested by Singer et al. [19].

The hardest histological differential diagnosis includes orbital teratoma, in which derivatives of all three germ lines are present [20]. Teratoma of the orbit may present with severe proptosis and rapid growth and malignant transformation is possible. Further differential diagnoses include microphthalmos with cyst, heterotropic brain tissue and meningoencephalocele. Microphthalmos with cyst is one of the colobomatous anomalies of ocular development which is derived from failed closure of the fetal cleft, resulting in a cyst attached to the sclera. Congenital cystic eye is less common than microphthalmos with cyst and it is similar to the cystic portion of micropthalmos with cyst, as reported by Dollfus et al. [21]. Meningoencephalocele is due to a defect of the cranio-orbital bones and the orbit may present with a cystic structure in the supero-medial canthal area, inducing proptosis [22]. True anophthalmia is extremely rare, although at birth the cystic eye may mimic anophtalmia [23].

There is no standardized protocol for management of the congenital cystic eye. Surgical intervention is strongly advised in order to obtain an optimal cosmesis. Regarding timing of surgery, cystic globes have been excised within a week to several years after the birth. After excision, Chaudry et al. [24] achieved acceptable cosmesis by fitting prosthesis, while Mansour et al. [25] performed the excision of a congenital cystic eye at the age of seven months without the use of an implant and the conjunctional fornix was fitted with progressively larger spheres. Robb et al. [26] reported a case of congenital cystic eye in which an initial attempt for excision was followed by recurrence of the cyst in the orbit after three months. Based on their experience, they concluded that every effort should be made to totally excise the congenital cystic eye when surgical removal is undertaken, due to the risk of recurrence.

At our knowledge, cerebellar lesion of supposed glial-origin has not been previously reported in the contest of the congenital cystic eye. Furthermore, spontaneous regression of low-grade glial lesions [27] is a rare but well recognized phenomenon [28], already described in children with [29] or without NF1 [30,31] and mainly after surgical intervention of biopsy/resection. Unfortunately, main limit of our description is the absence of the histopathologic characterization of the lesion in the cerebellum.

Brain tumors are the most common solid tumor diagnosed in childhood that account for significant morbidity and mortality. Children affected by low-grade gliomas are known to have an excellent 10-year survival rate. Bandopadhayay et al. [32] identified children diagnosed with low-grade gliomas between 1973 and 2008 through the Surveillance Epidemiology and End Results database. A total of 4040 pediatric patients aged <19 years were recorded (median age at diagnosis: 9 years, range 0–19 years; no gender difference). Pilocytic astrocytoma was the most common histology, accounting for 65% (Grade I: 74%, Grade II 26%). Supratentorial site(31%) and cerebellum (29%) were the preferential locations.

Therefore, in our case, the glial origin of the lesion could be anyhow suspected considering several elements. In pediatric population, the detection of incidental intracranial abnormalities can occur due to the advancing diagnostic imaging. Identified radiological features suggestive for low-grade gliomas are as follows [33]: non-enhancing, maximum diameter <1.5 cm, absence of surrounding edema. In these cases, treatment options include conservative strategies versus operative approaches and the choice is sometimes debated. The Joint Section on Tumors of the American Association of Neurological Surgeons/Congress of Neurological Surgeons has provided practice guidelines [34] that suggest that biopsy should be standard practice regardless of whether observation or further treatment is pursued. Conversely, Ali ZS et al. [35] presented a series of 12 pediatric patients with incidentally detected, small, non-enhancing, intracranial lesions that were treated conservatively with watchful waiting and closely serial MRIs. All patients were neurologically intact and the cerebellum was the most common lesion location. They proposed that the conservative approach offered a safe and effective alternative to invasive management. Non-operative management of incidental lesions radiologically consistent with low-grade gliomas could be favored because of the potential morbidity related to the technical aspects of the surgery, the anesthetic complications and the lack of sufficient data demonstrating improvement in the natural history of the disease. Moreover, significant limitations of diagnostic biopsy includes sampling error, particularly with small lesions located in the posterior fossa, with the risk of exposure to additional surgeries [36].

Low-grade glioma was also considered the most likely diagnosis in all cases, based on the consistent pattern of T1 hypointense, T2 and T2 fluid-attenuated inversion recovery hyperintense, and non-enhancing pattern of the lesions. Therefore, as in our case, in which the informed consent to the biopsy was denied, a “watchful waiting” management could be supported by the radiologic features consisting with a low-grade glial lesion and the absence of neurological deficits [37]. The interpretation of contrast enhancement is still debated [38]: in non-cerebellar gliomas, fluctuation of contrast enhancement during follow-up seemed to have no correlation with disease progression in absence of tumor size changes.

Biological mechanisms which induce spontaneous regression may only be speculated [39]. Several authors [40] hypothesize that indolent course and spontaneous regression of the lesion, predominantly in NF1 subset, could be related to a role of neurofibromin in down-regulation of RASA oncoprotein expression, that imply a decreased potentiality of cell proliferation. Furthermore, in patients ongoing biopsy or incomplete removal, it has been suggested that surgical intervention discloses previously protected antigens allowing a better recognition by the host’s immune system [41]. Moreover, surgical procedures seem to influence the growth attitude of the lesion by inducing vascular changes in the microenvironment and consequently leading tumor regression and reactive gliosis [42]. The expression of the apoptosis-related genes in the tumor [43], defined by well-know biologic markers as Apoptag, may also represent a contributing mechanism in the spontaneous involution.

## Conclusion

In conclusion, congenital cystic eye should be suspected in infants with an unrecognizable ocular globe. We present the first report of a low-grade lesion of suspected glial origin in the cerebellum, with unusual biological behavioral, disclosed in association with congenital cystic eye. Currently, we have no convincing explanation, supported by “ad hoc” literature and documentation of larger series, to explain the link between these two clinical entities; so it seems to be an accidental but very interesting association, in which two rare conditions are overlapped. We could also confirm that the well-known practice of early brain MRI in children with ocular malformations is very useful, in order to investigate the possible association with other intracranial alterations, also of non-malformative origin. This case presentation also highlights the radiologic features of the accidentally disclosed intracranial lesions that could justify a wait-and-see policy. Long-term follow-up is required to closely survey the tumor growth rate; in case of enhancement modifications, increase of size and/or edema appearance, biopsy and resection are then considered.

### Informed consent

Parental written informed consent was obtained for publication of this Case report and any accompanying images. A copy of the written consent is available for review by the Editor of this journal.

## Competing interests

All the authors disclose all potential, commercial, financial or other conflicting interests that could in any way affect the realization of this manuscript.

## Authors’ contributions

MGC wrote the first draft and cured the manuscript preparation. AR and AM cared for the patient. GSC selected the neuro-imaging and realized figure and legends. RDV selected and described the histopathological sections. AM has been crucial in ideating the work and supervising the draft revision. All authors read and approved the final manuscript.

## Pre-publication history

The pre-publication history for this paper can be accessed here:

http://www.biomedcentral.com/1471-2415/14/80/prepub

## References

[B1] Mann I A case of congenital cystic eye Trans Ophtalmol Soc Aust 1939 1 120 124

[B2] Shields JA Shields CL Orbital cysts of childhood–classification, clinical features, and management Surv Ophthalmol 2004 49 281 299 15110666 10.1016/j.survophthal.2004.02.001

[B3] Sacks JG Lindenberg R Efferent nerve fibers in the anterior visual pathways in bilateral congenital cystic eyeballs Am J Ophthalmol 1969 68 691 695 4186527 10.1016/0002-9394(69)91254-9

[B4] Hayashi N Repka MX Ueno H Iliff NT Green WR Congenital cystic eye: report of two cases and review of the literature Surv Ophthalmol 1999 44 173 179 10541156 10.1016/s0039-6257(99)00084-3

[B5] McLean CJ Ragge NK Jones RB Collin JR The management of orbital cysts associated with congenital microphthalmos and anophthalmos Br J Ophthalmol 2003 87 860 863 12812886 10.1136/bjo.87.7.860 PMC1771749

[B6] Shastry BS Persistent hyperplastic primary vitreous: congenital malformation of the eye Clin Experiment Ophthalmol 2009 37 884 890 20092598 10.1111/j.1442-9071.2009.02150.x

[B7] Tsitouridis I Michaelides M Tsantiridis C Spyridi S Arvanity M Efstratiou I Congenital cystic eye with multiple dermal appendages and intracranial congenital anomalies Diagn Interv Radiol 2010 16 116 121 19847771 10.4261/1305-3825.DIR.2054-08.1

[B8] Morselli PG Morellini A Sgarzani R Ghi T Galassi E Congenital cystic eye: from prenatal diagnosis to therapeutic management and surgical treatment J Craniofac Surg 2011 22 360 363 21239937 10.1097/SCS.0b013e3181f814dc

[B9] Gupta R Seith A Guglani B Jain TP Congenital cystic eye: features on MRI Br J Radiol 2007 80 e137 e140 17704309 10.1259/bjr/31817019

[B10] Duke-Elder S Duke Elder S Normal and Abnormal Development: Congenital Deformities System of Ophthalmology, Vol III. Part 2 1963 Henry Kimpton: London 451 481

[B11] Guthoff R Klein R Lieb WE Congenital cystic eye Graefes Arch Clin Exp Ophthalmol 2004 242 268 271 14676958 10.1007/s00417-003-0820-8

[B12] Dash RG Boparai MS Pai P Congenital ectopic encysted eye ball (a case report) Indian J Ophthalmol 1984 32 247 248 6443780

[B13] Shukla Y Kulshrestha OP Bajaj K Congenital cystic eye–a case report Indian J Ophthalmol 1984 32 249 250 6443781

[B14] Goldberg SH Farber MG Bullock JD Crone KR Ball WS Bilateral congenital ocular cysts Ophthalmic Paediatr Genet 1991 12 31 38 1881653 10.3109/13816819109023082

[B15] Raina UK Tuli D Arora R Mehta DK Bansal R Congenital cystic eyeball Ophthalmic Surg Lasers 2002 33 262 263 12027114

[B16] Pasquale LR Romayananda N Kubacki J Johnson MH Chan GH Congenital cystic eye with multiple ocular and intracranial anomalies Arch Ophthalmol 1991 109 985 987 2064582 10.1001/archopht.1991.01080070097044

[B17] Gupta VP Bhattacharya SN Gupta P Gupta R Is it congenital cystic eye with dermal appendages and cerebral anomalies? Indian J Ophthalmol 2010 58 448 20689212 10.4103/0301-4738.67053 PMC2992934

[B18] Mehta M Pushker N Sen S Sharma S Gupta K Bajaj MS Ghose S Congenital cystic eye: a clinicopathologic study J Pediatr Ophthalmol Strabismus 2010 23 47 10.3928/01913913-20100818-13 21158367

[B19] Singer JR Droste PJ Hassan AS Congenital cystic eye in utero: novel prenatal magnetic resonance imaging findings JAMA Ophthalmol 2013 131 1092 1095 23807213 10.1001/jamaophthalmol.2013.328

[B20] Gündüz K Kurt RA Heper AO Eye-conserving treatment in massive congenital orbital teratoma Clin Experiment Ophthalmol 2009 37 320 323 19459873 10.1111/j.1442-9071.2009.02024.x

[B21] Dollfus MA Marx P Langlois J Clement JC Forthomme J Congenital cystic eyeball Am J Ophthalmol 1968 66 504 509 5676359 10.1016/0002-9394(68)91537-7

[B22] Benharbit M Rifi LL El Khamlichi A Mohcine Z Orbital meningoencephalocele: two case studies J Fr Ophtalmol 2004 27 613 616 15343120 10.1016/s0181-5512(04)96186-4

[B23] Lowry RB Kohut R Sibbald B Rouleau J Anophthalmia and microphthalmia in the Alberta congenital anomalies surveillance system Can J Ophthalmol 2005 40 38 44 15825528 10.1016/S0008-4182(05)80115-2

[B24] Chaudhry IA Shamsi FA Elzaridi E Arat YO Riley FC Congenital cystic eye with intracranial anomalies: a clinicopathologic study Int Ophthalmol 2007 27 223 233 17453153 10.1007/s10792-007-9059-4

[B25] Mansour AM Li HK Congenital cystic eye Ophthal Plast Reconstr Surg 1996 12 104 107 10.1097/00002341-199606000-00004 8727176

[B26] Robb RM Anthony DC Congenital cystic eye: recurrence after initial surgical removal Ophthalmic Genet 2003 24 117 123 12789576 10.1076/opge.24.2.117.14002

[B27] Schmandt SM Packer RJ Vezina LG Jane J Spontaneous regression of low-grade astrocytomas in childhood Pediatr Neurosurg 2000 32 132 136 10867559 10.1159/000028917

[B28] Parsa CF Hoyt CS Lesser RL Weinstein JM Strother CM Muci-Mendoza R Ramella M Manor RS Fletcher WA Repka MX Garrity JA Ebner RN Monteiro ML McFadzean RM Rubtsova IV Hoyt WF Spontaneous regression of optic gliomas: thirteen cases documented by serial neuroimaging Arch Ophthalmol 2001 119 516 529 11296017 10.1001/archopht.119.4.516

[B29] Allen JC Initial management of children with hypothalamic and thalamic tumors and the modifying role of neurofibromatosis-1 Pediatr Neurosurg 2000 32 154 162 10867564 10.1159/000028922

[B30] Rozen WM Joseph S Lo PA Spontaneous regression of low-grade gliomas in pediatric patients without neurofibromatosis Pediatr Neurosurg 2008 44 324 328 18504420 10.1159/000134925

[B31] Gallucci M Catalucci A Scheithauer BW Forbes GS Spontaneous involution of pilocytic astrocytoma in a patient without neurofibromatosis type 1: case report Radiology 2000 214 223 226 10644128 10.1148/radiology.214.1.r00ja07223

[B32] Bandopadhayay P Bergthold G London WB Goumnerova LC Morales La Madrid A Marcus KJ Guo D Ullrich NJ Robison NJ Chi SN Beroukhim R Kieran MW Manley PE Long-term outcome of 4,040 children diagnosed with pediatric low-grade gliomas: an analysis of the Surveillance Epidemiology and End Results (SEER) database Pediatr Blood Cancer 2014 61 1173 1179 24482038 10.1002/pbc.24958 PMC4657506

[B33] Di Rocco C Frassanito P Tamburrini G The never-ending struggle between the two souls of the neurosurgeon: to wait or to intervene World Neurosurg 2014 81 268 270 23500336 10.1016/j.wneu.2013.02.073

[B34] Brown PD Wald JT McDermott MW Baumann GS Cloughesy TF Oncodiagnosis panel, patient’s symptoms not related to the lesion seen in the MR images Radiographics 2002 2003 23 1591 1611 14635615

[B35] Ali ZS Lang SS Sutton LN Conservative management of presumed low-grade gliomas in the asymptomatic pediatric population World Neurosurg 2014 81 368 373 23321383 10.1016/j.wneu.2013.01.038

[B36] Perret C Boltshauser E Scheer I Kellenberger CJ Grotzer MA Incidental findings of mass lesions on neuroimages in children Neurosurg Focus 2011 31 E20 22133179 10.3171/2011.9.FOCUS11121

[B37] Pollack IF Management of low-grade gliomas in childhood World Neurosurg 2014 81 265 267 23376380 10.1016/j.wneu.2013.01.104 PMC3949177

[B38] Gaudino S Quaglio F Schiarelli C Martucci M Tartaglione T Gualano MR Di Lella GM Colosimo C Spontaneous modifications of contrast enhancement in childhood non-cerebellar pilocytic astrocytomas Neuroradiology 2012 54 989 995 22286205 10.1007/s00234-012-1010-3

[B39] Ogiwara H Bowman RM Tomita T Long-term follow-up of pediatric benign cerebellar astrocytomas Neurosurgery 2012 70 40 47 21808215 10.1227/NEU.0b013e31822ff0ed

[B40] Piccirilli M Lenzi J Delfinis C Trasimeni G Salvati M Raco A Spontaneous regression of optic pathways gliomas in three patients with neurofibromatosis type I and critical review of the literature Childs Nerv Syst 2006 22 1332 1337 16639629 10.1007/s00381-006-0061-3

[B41] Kernan JC Horgan MA Piatt JH D’Agostino A Spontaneous involution of a diencephalic astrocytoma Pediatr Neurosurg 1998 29 149 153 9838268 10.1159/000028710

[B42] Bilginer B Narin F Oguz KK Uzun S Soylemezoglu F Akalan N Benign cerebellar pilocytic astrocytomas in children Turk Neurosurg 2011 21 22 26 21294087

[B43] Steinbok P Poskitt K Hendson G Spontaneous regression of cerebellar astrocytoma after subtotal resection Childs Nerv Syst 2006 22 572 576 16552566 10.1007/s00381-006-0058-y

